# Physically Active Play as a Context for Motor Learning in Children with and Without Developmental Coordination Disorder: A Conceptual Synthesis of Cross-Domain Alignment

**DOI:** 10.3390/children13060723

**Published:** 2026-05-22

**Authors:** Osnat Atun-Einy, Maninderjit Kaur

**Affiliations:** 1 Department of Physical Therapy, Faculty of Social Welfare and Health Sciences, University of Haifa, Haifa 3103301, Israel; oatun@univ.haifa.ac.il; 2 Physical Therapy Department, School of Health and Rehabilitation Sciences, MGH Institute of Health Professions, Charlestown, MA 02129, USA

**Keywords:** children, active play, physically active play, motor learning, neurodevelopmental disorder, developmental coordination disorder

## Abstract

Background/Objectives: Physically active play (PA-play) offers natural, self-directed, and varied opportunities for physical activity and motor skill development in children. It is often viewed as a rich context for learning, yet how PA-play systematically supports the core concepts and elements of motor learning (ML) requires a closer examination in typically developing (TD) children and those with neurodevelopmental disorders such as Developmental Coordination Disorder (DCD). Methods: A two-part integrative conceptual synthesis was conducted to explore how core ML concepts are reflected in children’s PA-play. Part 1 involved a synthesis of the play literature, analyzed through an ML lens in TD children. Part 2 involved synthesizing the literature on ML elements and characteristics of PA-play in children with DCD. Results: In Part 1, the conceptual synthesis highlighted that PA-play in TD children enables conditions supportive of ML, including both implicit and explicit learning, high-volume practice, task variability, progressive challenge, and feedback through verbal and non-verbal cues. In Part 2, the synthesis highlighted ML difficulties in children with DCD, such as slow, effortful learning with reduced adaptability and greater performance variability. Additionally, the synthesis highlighted limited DCD evidence using PA-play as an ecological context for ML. Conclusions: Overall, PA-play could offer environments consistent with ML elements in TD children, yet evidence for its effectiveness in children with DCD remains limited. Future research should explore how PA-play can be leveraged to address the specific ML challenges faced by children with DCD.

## 1. Introduction

### 1.1. Motor Development and Motor Learning

Motor development is a foundational aspect of childhood, providing the basis for action, interaction, and learning [1]. It begins with the acquisition of early milestones, such as crawling and walking, followed by fundamental skills in locomotion, object interaction, and stability/balance, which then progress to complex, culturally specific activities in play, sport, and daily life [2,3,4,5]. Motor development is deeply intertwined with other developmental domains, such that advancements in motor abilities can improve cognition, communication, and social participation [6,7]. Conversely, development in these other domains further refine motor ability, highlighting their reciprocal and ongoing interplay [8]. These developmental processes are embedded within environmental contexts, where physical and social affordances at home, school, and neighborhood shape opportunities for motor engagement and learning [9].

One key process driving developmental progression is motor learning (ML). It is a lifelong process involving the acquisition of new movement patterns and the sensorimotor adaptation of existing ones, with retention and transfer serving as key indicators of learning success [10,11]. ML has attracted broad interdisciplinary interest, with various theories, including Schmidt’s schema theory [12] and dynamic systems/ecological approaches [13,14,15], emphasizing the role of practice variability, task demands, and the environment in shaping learning. Additionally, motivational factors and psychological needs, such as self-efficacy, autonomy, and social relatedness, as emphasized in Self-Determination and OPTIMAL theories, influence the learning process [16,17].

During childhood, ML is particularly dynamic, as children acquire a vast range of skills for the first time. This process is also influenced by the rapid physical growth and maturation of body systems, which further shape how skills are acquired and refined [10,18]. Moreover, ML varies significantly depending on children’s diverse abilities and needs, including those with neurodevelopmental disorders such as Developmental Coordination Disorder (DCD) [18,19,20,21]. For example, children with DCD often require more practice and support to acquire motor skills compared with typically developing (TD) peers [18,22]. Lastly, ML in children occurs across a range of structured and unstructured contexts that vary throughout development [23,24]. Given this multifaceted nature, the present synthesis aims to provide a comprehensive integrative perspective of ML in both TD children and those with DCD, within the context of physically active play.

### 1.2. Physically Active Play in Children

Play, in its many forms, is an intrinsically motivated, self-directed, enjoyable, and process-oriented activity that supports development across motor, cognitive, social, emotional, and language domains [25,26,27,28,29,30,31,32]. The current synthesis focuses on physically active play (also known as active play, physical play, locomotor play, gross motor play, exercise play, and movement play) as it relates to motor development and ML. At its core, physically active play (hereafter referred to as PA-play) involves large whole-body movements (e.g., running, racing and chasing, jumping, climbing, spinning, and throwing and catching) resulting in physical effort and energy expenditure above resting levels [33,34,35,36,37]. It involves different forms, such as free/unstructured play, rough-and-tumble play, rhythmic movement games, and play with rules or imaginative elements that can vary with the age and developmental status of the child [34,35,37,38,39,40]. It may occur alone or in conjunction with others, across various locations, both indoors and outdoors, in natural and urban environments [34,36]. While universal, its meaning and form can vary across cultures, for example, in traditional folk games and street plays [34,41]. For the current manuscript, PA-play will be discussed in general terms, as a developmental context, without differentiating its specific forms or associated terminology.

In the literature, PA-play is usually recognized with regard to its contribution to promoting motor development and physical activity in children, and its pedagogical value in early education. The role of PA-play in promoting motor development and physical activity among children is widely recognized, particularly in light of growing concerns about sedentary behaviors and the increasing prevalence of childhood obesity [36,42,43]. PA-play complements other forms of physical activity, such as organized sports and active commuting, in meeting recommended daily activity guidelines. Additionally, the health-related benefits of PA-play are well documented [36,44,45,46], along with its role in promoting fundamental movements of children [45,47,48,49,50]. Through PA-play, children also test their physical abilities and gain a sense of mastery over physical challenges, supporting body awareness, spatial boundaries, attention, and self-regulation [26,37]. These experiences contribute broadly to children’s well-being, fostering physical, cognitive, social, and psycho-emotional growth [28,29,51,52]. PA-play in educational settings promotes physical, academic, and social–emotional development, including creativity, resilience, and social collaboration [38,53]. Such benefits are increasingly documented in outdoor programs like forest kindergartens, which use natural settings to foster experiential learning and broader educational objectives [54].

### 1.3. The Intersection of Motor Learning in the Context of Physically Active Play

The role of PA-play in supporting motor development and physical activity, as outlined above, is well recognized [36,42,43,44]. Similarly, PA-play (or the broader concept of play) has been studied with respect to one or more ML elements, such as skill acquisition, practice, and transfer [45,47,48,49,55]. Despite these contributions, the relationship between PA-play and ML has not been systematically synthesized, and the ways in which PA-play characteristics align with each core ML element remain underexplored. A comprehensive conceptual integration of how PA-play characteristics align with and potentially support diverse ML processes is lacking. This gap limits our understanding of how motor learning unfolds in naturalistic contexts such as PA-play, complementing existing recommendations to promote PA-play for physical, cognitive, and psychological benefits [28,51,52]. Addressing this gap is particularly important for children with typical development and for those with DCD.

### 1.4. The Current Conceptual Synthesis

To address the literature gaps outlined above for both typically developing children and those with DCD, the current study examines the alignment between PA-play and ML. Given the broad, multidisciplinary nature of both fields and the conceptually oriented questions guiding this work, we adopted a conceptual synthesis approach [56,57], a method within the spectrum of integrative reviews that organizes and interprets complex cross-disciplinary evidence [56]. This approach enables and facilitates the identification of meaningful conceptual links and gaps that may not be apparent through systematic or outcome-focused reviews, which are less suited to capture connections across such a diverse body of literature [56,58,59,60,61].

The conceptual synthesis of cross-domain alignment first focuses on typically developing children, for whom extensive research exists on PA-play and ML, although these areas have rarely been examined together. The second stage of the conceptual synthesis examines children with DCD, a population selected for its well-documented unique ML challenges and play characteristics [18,22,62,63]. This study’s contribution lies in organizing and integrating knowledge across domains and populations, rather than generating new empirical data.

### 1.5. Research Questions

This study addresses the following research questions: 1. How do the characteristics of PA-play align with the core elements of ML, as defined by the ML framework in TD children? 2. How might the conceptual alignment between PA-play and ML, as observed in TD children, be applied to children with DCD?

## 2. Methods

### 2.1. Methodological Approach

The present study is positioned as a conceptually driven synthesis, aiming to integrate and align constructs across two domains: PA-play and ML, in two populations: TD children and children with DCD. This approach draws on principles of integrative and conceptual synthesis of the literature, emphasizing the organization and synthesis of knowledge to clarify relationships between constructs across disciplines, typically drawing on multiple concepts, literature streams, and theories [56,57,61]. The synthesis is interpretive and integrative, highlighting conceptual correspondence rather than empirically testing or validating causal relationships, as detailed below. The conceptual synthesis of a cross-domain alignment approach (hereafter referred to as the ‘synthesis’) is well suited to addressing the research questions, as it facilitates the integration of diverse research findings across sources and disciplines, enabling the conceptual alignment of key constructs. This approach prioritizes the conceptual relevance of the literature over exhaustive coverage, with literature selection being guided by its relevance to aligning the cross-domain constructs (specifically PA-play and ML), rather than completeness [56,57]. Given the extensive and theoretically diverse nature of the literature on ML and play across multiple disciplines, systematic aggregation is neither feasible nor suitable [58,64,65]. Additionally, the study incorporates the literature from two distinct populations: TD children and those with DCD. This targeted integration provides valuable insights that a systematic review would not offer, particularly given the focus on cross-domain connections rather than an outcome-focused aim.

### 2.2. Motor Learning and Play Terminology Considerations

Within conceptual synthesis, addressing variability in terminology and conceptual definitions and frameworks is a necessary methodological step to enable coherent cross-domain integration [56].

The literature on ML spans multiple disciplines and shows considerable variability in how ML elements are defined and described [20,66,67,68,69]. To manage this complexity, and ensure a coherent presentation of ML elements, it was necessary to select an organizational structure to chart ML elements and establish alignment with PA-play. We used the organizational structure of ML elements outlined by Kafri and Atun-Einy [66] in their scoping review, which mapped 12 well-established ML theoretical frameworks with a practical, clinically oriented focus to guide clinicians in applying ML principles in practice. Importantly, this structure does not introduce any new elements, definitions, or theoretical constructs. The work was expanded in subsequent work [70], which provides detailed explanations of individual ML elements. The above works, co-authored by the first author of the current study, serve solely as a methodological tool to organize and chart core ML elements, not as a source of evidence in the interpretation or synthesis of the literature. Therefore, while it presents a specific framing of ML from a clinical perspective rather than a broader conceptual perspective, apart from this contextual focus, it does not introduce any other bias into the recommendations or synthesis presented in this manuscript.

Within this organizational structure, the key components of ML, which are well established in the literature, are categorized into: (a) general learning concepts (e.g., stage of learning and implicit vs. explicit learning), (b) practice variables (e.g., amount and whole vs. part practice), (c) task presentation (e.g., mode of instructions), (d) psychological dimensions (e.g., motivation and self-efficacy), and (e) specific learning strategies (e.g., observational learning). Although there is some overlap of learning strategies covered in (a) general learning concepts and (e) specific learning strategies, we choose to discuss them as distinct categories. The rationale for this decision is to align with the interdisciplinary literature on this topic, including physical therapy, occupational therapy, physical education, and sports science. Learning strategies are at the center of this interdisciplinary literature, ranging from educational to clinical to empirical studies in children with and without DCD. Therefore, ML strategies are discussed both as overarching theoretical principles and as approaches tailored to specific learning styles [67,68,71].

In the context of the play literature, synthesizing the literature on play presents unique challenges due to its conceptual and terminological complexity. Play is a multidimensional construct, which includes various types, purposes, and contexts, and it is studied across multiple disciplines using overlapping terminology [72,73]. This complexity extends to PA-play, which could be described as ‘active play’ and ‘gross motor play,’ or embedded within broader categories, such as ‘free play,’ ‘outdoor play,’ or ‘nature play’ without being explicitly labeled in titles, abstracts, or indexing terms [74]. Many of these terms overlap, and some combine multiple play concepts, making relevant studies difficult to identify. As a result, narrowly defined search terms may exclude important research [75]. To address this, we included studies that explicitly examined PA-play as well as broader play research that was conceptually relevant to the active play context. Broader sources were only included when their analyses, examples or discussions directly referred to PA-play, ensuring that all the included literature contributed meaningfully to the synthesis.

### 2.3. Literature Search and Selection Strategy

In alignment with our conceptual synthesis approach, we adopted a flexible and iterative search strategy, which differed from the exhaustive data collection typical of systematic reviews. Rather than focusing solely on comprehensive coverage and statistical analysis, our goal was to highlight the conceptual connections between ML and PA-play. To achieve this, we employed keyword-based database searches and the snowballing technique, practices commonly accepted for discursive synthesis of broad, heterogeneous research [76,77]. The eligibility criteria for inclusion of sources were (1) sources published in English between 1998 and 2025, (2) sources providing description or data relevant to PA-play or ML in TD children and those with DCD, and (3) peer-reviewed articles, book chapters, reviews, or theoretical/perspective papers offering theoretical, empirical, or perspective-based insights useful for cross-domain synthesis, capturing the breadth of the field. Conversely, the exclusion criteria of this study were (1) sources not published in peer-reviewed journals, except selected scholarly book chapters from reputable academic publishers, including university presses and specialist scholarly publishers (e.g., Elsevier, Harvard University Press, Human Kinetics, Guilford Press, and MIT Press); (2) non-English sources; (3) studies not focused on ML elements or PA-play as defined in the current study, for example, studies on video/computer games (screen-based activities); and (4) studies involving populations other than TD children or those with DCD.

**Figure 1:**
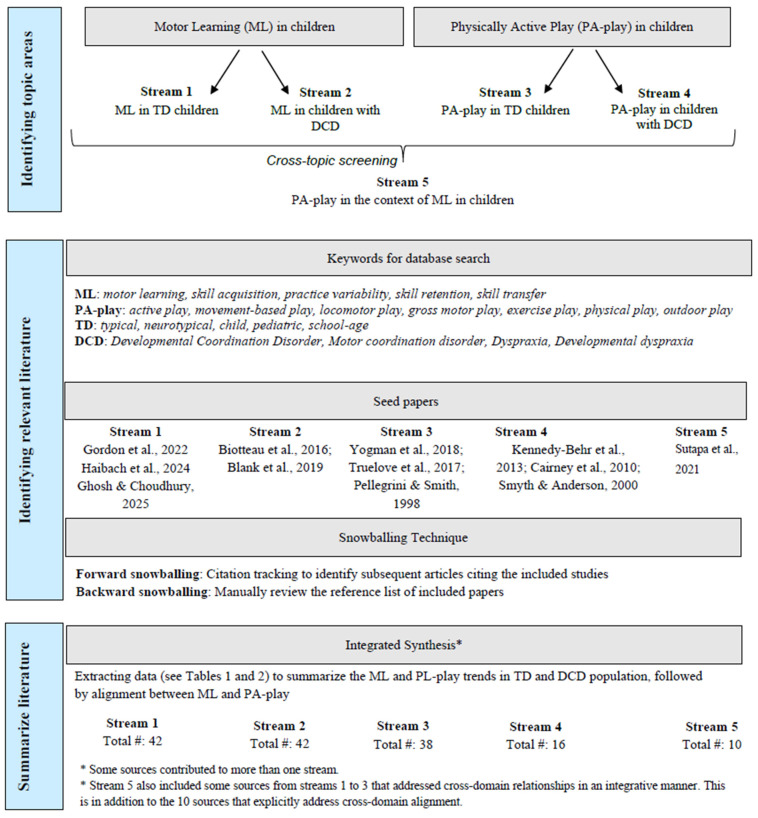
Flow diagram of the literature search and selection process [18,19,22,23,31,36,37,63,78,79,80,81].

Our database search was focused on two parallel topics, PA-play and ML, which were further subdivided based on the population, i.e., TD children and those with DCD. Additionally, a cross-topic stream (PA-play × ML) was included, resulting in a total of 5 different search streams (see Figure 1). Keywords were identified for each search stream, acknowledging that PA-play keywords required flexibility (see Section 2.2 for details). Next, purposive searches were conducted across major databases (i.e., PubMed, Google Scholar, and Scopus) to identify sources. Within each stream, an initial set of seed papers (i.e., systematic and scoping reviews) was identified, especially in the TD population, due to the broad scope of ML research. These seed sources also served as the basis for iterative snowballing, including backward (reference list screening) and forward (citation tracking) cycles, as well as targeted searches for studies addressing specific ML elements relevant to the ML adopted organizational structure, continuing until no new relevant information was identified [76].

Given our study’s goal of cross-domain alignment, initially, sources identified for stream 5 (PA-play × ML) were used. An iterative integrative synthesis was then conducted to extract, refine, and align findings across streams, allowing insights from streams 1 and 4 to inform the cross-domain analysis. Importantly, the aim of this synthesis was not to comprehensively review all the literature, but to identify points of alignment between PA-play and ML elements, documenting instances where support for alignment was evident or where no alignment was found. The research team’s prior knowledge additionally informed study selection and cross-domain alignment, which is consistent with established guidance on narrative synthesis of the literature [58,59,64,65].

‘Alignment’ refers to an interpretive, theory-informed mapping between characteristics of PA-play and ML elements. It was determined when descriptions of PA-play in the literature were found to be consistent with the underlying ML elements (e.g., repetitions and variability of practice). This process does not imply empirical validation or causal relationships but represents a narrative and conceptual correspondence based on convergence across literature sources. For example, the ML element ‘amount of repetitions’ was extracted from the PA-play literature explicitly describing the repeated execution of motor actions. These descriptions were expressed using various terms, including repeated trials, ongoing practice, repeated experimentation, movement repetition, and motor training embedded within play activities. All highlight that children naturally engage in repetitive motor behavior during PA-play, demonstrating alignment with the ML element of amount of practice or repetitions. Similarly, PA-play characterized by spontaneous variation in movement contexts was interpreted as aligning with the ML principle of practice variability, based on theoretical definitions of variability in the ML literature.

Figure 1 presents the flow diagram of the literature search and selection process. It illustrates the five search streams and the process of searching, identification, and inclusion and reports only the number of studies included in each stream. Note that the figure does not provide quantitative counts of records identified, screened, or excluded at each stage, as such detailed enumeration is beyond the scope of a conceptual synthesis [78,82]. The figure is intended to emphasize the key steps of the search and selection process and the structure of the included studies, rather than to imply full reproducibility.

## 3. Analytical Synthesis

### 3.1. Overview of the Analytical Synthesis

This section presents an analytical synthesis of the proposed conceptual cross-domain alignment. The purpose is to structure and articulate cross-domain alignment rather than to provide empirical demonstration. In Part 1 of the synthesis, we first summarize the literature on ML in TD children using the ML terminology and organizational structure indicated above [66]. This is followed by a review of the PA-play literature and aligning the characteristics of PA-play in TD children with the core elements of ML. In Part 2 of the synthesis, due to the limited research on the alignment between PA-play and ML in children with DCD, a direct comparison to the alignment done in TD children is not possible. Instead, we begin with a brief description of the condition, followed by a summary and an overview of the ML elements and PA-play characteristics in children with DCD.

### 3.2. Alignment of Motor Learning in the Context of Physically Active Play in Typically Developing Children (Part 1)

In this section, we present the alignment between ML elements and PA-play characteristics in TD children. While the synthesis maps and organizes correspondences between PA-play and ML elements, it does not empirically test or demonstrate causal or functional effects. Here, ‘alignment’ refers to an interpretive and integrative mapping of existing evidence, highlighting conceptual relationships rather than validating them. Table 1 summarizes the ML elements and their alignment with PA-play in TD children. The table is organized into three columns: The first lists the ML elements, organized according to the framework described earlier [66,70]. The second summarizes the ML trends in TD children, while the third outlines the alignment between PA-play and each ML element.

As detailed in Table 1, the analysis of the evidence indicates that while some ML elements show clear and direct alignment with the characteristics of PA-play, others do not demonstrate specific or consistent alignment. The following sections summarize these findings, highlighting both the alignments and the gaps identified in the data.

*From general learning concepts*, elements such as ‘meaningful/salient goals’, ‘active engagement’, and ‘progression of task difficulty’ are found to align with PA-play characteristics. Drawing on multiple sources (see Table 1), PA-play is consistently described as a meaningful activity, simulates active participation, and presents varied task challenges to children, whereas limited supporting sources indicate that general concepts such as ‘learning mechanism’ and ‘classification of motor skills’ are only partially supported by PA-play, as children engage in different types of motor skills during play, and their actions are not bound to a structured classification system. The element ‘stage of skill acquisition’ does not demonstrate strong alignment with PA-Play, as children are at different stages of skill acquisition during play.

*Regarding practice variables*, ‘specificity’ and ‘positive reinforcement’ align with PA-play. Other practice variables, such as ‘amount of practice’, ‘practice variability’, ‘task breakdown’, and ‘feedback’, are partially supported by PA-play, as PA-play includes opportunities to practice and receive feedback for a variety of skills, but not by using a structured classification system. ‘Practice schedule’ is not aligned with PA-play, as children do not follow a specific practice schedule.

*In terms of task presentation*, ‘mode of instruction’ and ‘instruction’s direction of attention’ do not align with PA-play, as children use different modes of instruction and their attentional focus involves both internal and external focus. *In the psychological domain*, ‘intrinsic motivation and related affective and social–cognitive factors’ align with PA-play, as play is often described as fun/enjoyable, and it offers abundant opportunities to explore and experiment. Similarly, the learning strategy of ‘trial-and-error learning’ aligns with PA-play characteristics, whereas learning strategies such as ‘discovery learning’, ‘motor imagery’, and ‘analogy learning’ show partial alignment. ‘Errorless learning’ is not aligned with PA-play, as children perform and learn from errors during play.

### 3.3. Exploring Motor Learning in the Context of Physically Active Play in Children with Developmental Coordination Disorder (Part 2)

Given the limited research on the alignment of PA-play with ML in children with DCD, a full alignment between PA-play and ML elements, as seen in the table for TD children, could not be made. Instead, we begin with a general overview of DCD, followed by a focus on the ML challenges and PA-play characteristics specific to children with DCD. In Table 2, we summarize the key ML elements for children with DCD and provide interpretive notes based on the PA-play characteristics observed in TD children.

DCD is a neurodevelopmental condition characterized by an impairment in the planning and execution of movement that interferes significantly and persistently with activities of daily life, academic/school performance, and participation in age-appropriate tasks of leisure and play. Symptoms typically emerge in early childhood and are diagnosed when motor challenges persist despite adequate learning opportunities and cannot be explained by other medical or developmental conditions [182,183]. DCD affects around 5–6% of children, though rates vary by country, with higher prevalence in males. Though the etiology of the disorder remains unclear, it is linked to differences in activation in several neural mechanisms involved in motor planning and control [183,184]. Presentations vary widely, with variability in motor symptomatology, severity, and co-occurring conditions. Secondary impacts of DCD often include psychosocial challenges such as low self-esteem, and self-efficacy, and withdrawal from social and physical activities, as well as related health issues like obesity and poor physical fitness [185,186].

#### 3.3.1. Motor Learning in Children with Developmental Coordination Disorder

Table 2 summarizes the ML elements for the DCD population. In terms of general learning concepts, children with DCD demonstrated delayed and more effortful progression through the stages of motor skill acquisition compared with TD peers, often relying on compensatory strategies that were less efficient [18,22,146]. This is accompanied by increased inter-trial variability in movement kinematics and kinetics [144,157]. Similarly, evidence for implicit versus explicit learning remains mixed, with some studies suggesting impairments in implicit learning and others reporting intact performance [148,149]. In terms of practice variables, children with DCD required greater practice dosage, including extended repetitions and structured task support, to reach performance levels comparable to those of TD peers [22,144]. Furthermore, increased practice repetition alone was insufficient, without opportunities for problem solving and task-specific training. Feedback studies indicated an over-reliance on external visual feedback, with promising effects for self-controlled and technology-assisted approaches, though no single feedback type has proven superior [162,165]. In terms of psychological dimensions, motivation emerged as a significant barrier to ML in children with DCD, with repeated failures contributing to low self-efficacy and avoidance of motor tasks [169,172]. Lastly, for learning strategies, observational learning and motor imagery appeared less effective in isolation but could be enhanced when combined [176,178]. Analogy learning and errorless learning show potential but remain under-researched [147]. See Table 2 for details.

The ML difficulties of children with DCD could be understood based on theoretical frameworks. Children with DCD have deficits in internal modeling, which limits predictive control, error-based learning, and anticipatory planning [141,187]. As a result, children rely more on slower, feedback-driven, reactive strategies rather than predictive control. Internal modeling deficits are evident across effector systems, including oculomotor, fine manual tasks, and dynamic postural control [157,187]. Through the lens of schema theory, internal models are analogous to recognition schemas that predict sensory outcomes, while the generation of motor commands is analogous to recall schemas that guide movement selection and adjustment [12]. From a dynamic systems and ecological perspective, variability is functional, allowing for self-organization, flexibility, and adaptability. In early stages of ML, high variability allows for the exploration of a wide repertoire of coordination solutions, which typically stabilize into efficient patterns with practice [188,189,190]. In children with DCD, unique constraints can lead to compensatory movement solutions, and they may struggle with functional variability. For example, excessive or atypical variability may disrupt the learning process, slow skill acquisition, and reduce adaptability across tasks [146,191].

#### 3.3.2. Motor Learning in the Context of Physically Active Play in Children with Developmental Coordination Disorder

A growing body of research is attempting to incorporate playful or whole-body elements into ML interventions for children with DCD, including the use of virtual reality video games (e.g., Wii Fit) and activity-based, problem-solving tasks [21,192,193,194,195]. Additionally, play-based components are often integrated into task-specific interventions, such as Neuromotor Task Training [142], or group interventions [196], reflecting the growing recognition of the value of play in enhancing engagement and facilitating positive outcomes in motor skill acquisition and transfer. Nevertheless, studies that examine ML as it naturally unfolds within real-world play contexts, particularly in children with DCD, remain limited. The integration of ML into real-world PA-play, where learning is embedded in the dynamic and interactive environments of children, is still rare. Therefore, this section focuses on the characteristics of PA-play in children with DCD, with particular attention to how these differ from those observed in TD peers.

Children with DCD often demonstrate limited engagement in physical activity, including PA-play, spending less time in moderate to vigorous activity compared with their peers, and favoring screen-based tasks over physical play [197,198,199]. In preschool, children with DCD tend to prefer non-motor-based play, exhibit more onlooker behavior, and spend more time transitioning between activities rather than engaging in sustained play [62,63]. Moreover, when preschoolers engage in play, they demonstrate less mature play patterns, including reduced physical construction and social play, compared with typical peers [62,63,200]. As children grow older, disparities in PA-play participation become more pronounced. School-aged children with DCD are more likely to observe rather than engage in physical games, report lower enjoyment, and tend to choose less demanding, non-motor-based activities during recess or free time [80,81,201]. On the school playground, they often spend time alone, show reduced participation in large-group physical games, and engage less in social physical play overall. However, their involvement in social fantasy play remains similar to that of TD peers [81].

These patterns of play behaviors in the DCD population are initially shaped by repeated experiences of motor difficulty, such as an increased risk of falls and frequent missed targets, and are further complicated by negative attitudes towards play. As a result, they often withdraw from active play, further reducing opportunities to practice and refine their motor skills [202]. As children begin to compare themselves to others from around age five, early challenges in motor tasks can significantly influence their motivation and attitudes toward physical play [22,185,201,203]. This developmental trajectory reflects the *activity deficit hypothesis*, which suggests that poor motor skills lead to reduced motivation and physical activity engagement, thereby reinforcing motor challenges [204]. Ultimately, this cycle continues as reduced engagement in PA-play, including other physical and leisure activities, reinforces a pattern of motor underdevelopment, low self-efficacy, and social withdrawal in children with DCD [169,201,203,205].

Table 2 presents, in the first column, the core ML elements, followed by their observed manifestations in children with DCD in the second column. As it was not possible to establish a research-based alignment between ML and PA-play as done in Table 1, the third column, titled ‘PA-Play-Related Notes Based on Typical Development’, provides interpretive insights into potential connections between the characteristics of PA-play and ML challenges in children with DCD. These notes are not derived from DCD-specific research and, as such, do not aim to provide a direct alignment. Instead, they are based on the characteristics and potential benefits of PA-play observed in TD children. It offers conceptual directions, with a focus on selected ML elements that exhibit a conceptual link to PA-play.

The table highlights several key points regarding ML in children with DCD. Progress in skill acquisition typically requires more time, repetition, and support, often relying on compensatory strategies that are less efficient. Goal setting aligned with the child’s interests enhances motivation and engagement, but structured support is essential due to motor difficulties and low self-efficacy. In terms of practice, increased repetitions, structured variability, and tailored feedback (both internal and external) are critical to improving motor skills. Psychological factors, such as reduced motivation and self-efficacy, often hinder participation and engagement. Additionally, strategies like motor imagery and errorless learning have shown promising results, and guided, discovery-based approaches are beneficial. However, further research is needed to identify the most effective strategies for this population.

## 4. Discussion

The current synthesis aimed to integrate research at the intersection of ML and PA-play in children with and without DCD, providing a structured conceptual perspective on cross-domain relationships through an interpretive and integrative approach. Although both ML and PA-play are well-established research areas across multiple disciplines, they have largely been studied in isolation, with little focus on how ML principles may naturally emerge within everyday play contexts. The novelty of this synthesis lies in highlighting the alignment between these two broad domains, illustrating aspects of PA-play as a dynamic, ecologically valid context for ML in children. Importantly, this work should be understood as a conceptual and interpretative synthesis rather than an empirical or demonstrative analysis and therefore does not aim to establish replicability in the experimental sense. Our key observations indicate that PA-play provides a developmentally rich context in which some core ML elements, including intrinsic motivation, repetition with variability, progressive challenge, and social learning, are naturally embedded, albeit in fluid and non-linear ways that differ from traditional structured practice. Importantly, while this alignment appears robust in TD children, a significant gap remains in understanding how ML unfolds within naturalistic PA-play contexts in children with DCD, where research continues to rely predominantly on laboratory-based or highly controlled tasks. In the following sections, we provide a detailed discussion of the results from the TD population (Section 4.1), DCD population (Section 4.2), and clinical implications for key stakeholders (Section 4.3).

### 4.1. Physically Active Play as a Context for Motor Learning in Typically Developing Children

The reviewed literature establishes PA-play as a highly versatile and developmentally rich context for applying ML elements in TD children. Included studies explored different types of PA-play in TD children, which span over a continuum from spontaneous, child-led free play to more structured peer- or adult-led play. Regardless of the variability in play types, several core ML characteristics consistently emerge during play. At its core, PA-play is described as being driven by enjoyment and freedom of choice, factors that promote deep engagement [31,39,55,85,86,87,88,92]. When their voices are included, children consistently emphasize free choice and autonomy, friendships, and opportunities for meaningful, often outdoor play [28,53,92]. These play characteristics closely align with the psychological dimensions of ML as described in Self-Determination [16] and the OPTIMAL theory of ML [17]. In children, findings from a scoping review suggest that OPTIMAL variables also support effective ML. However, developmental differences may alter these effects, underscoring the need for further research [116]. Furthermore, PA-play naturally provides joyful and engaging experiences that involve active bodily participation and rich sensorimotor interaction with the environment [40]. Children engage in dynamic, multi-sensory experiences that naturally foster children’s motor exploration, adaptation, and problem solving [29,31,51,79,99], processes which are fundamental to ML [19].

As highlighted in Table 1, several ML elements align well with PA-play in TD children. Therefore, PA-play can be considered a uniquely effective setting for learning and refining motor skills. The movement opportunities afforded by PA-play are developmentally appropriate and tailored to the child’s individual interests and abilities [19,38,55]. During PA-play, children are physically, mentally, and emotionally invested, often entering a profound state of immersion where they become fully absorbed in the activity. This immersive state, marked by high enjoyment and focused attention, overrides perception of forced effort and fatigue, which might be encountered during other daily activities. Consequently, children can engage in sustained and spontaneous repetitions, a critical element of ML. These repetitions are driven by intrinsic motivation rather than external demands [31,36].

Additionally, as children engage in repeated actions during PA-play with subtle or substantial variations in force, timing, or spatial parameters, they generate movement variability. This variability of practice is again fundamental to effective ML [104,206]. Such variability supports the discovery and stabilization of adaptable movement patterns [13,71]. PA-play offers plentiful and cumulative opportunities for children to practice the same motor skills across diverse environments and materials. Through these repeated variations, children explore multiple movement solutions, enabling progressive refinement through sustained, self-directed experimentation [28,31,51,99,104]. Another element that PA-play fosters naturally is the gradual progression of task difficulty and challenge. Progression emerges organically during PA-play and helps master increasingly complex tasks. Moreover, the evolving rules of traditional and modern motor games, along with the interactions with play partners, actively contribute to the gradual increase in difficulty. Together, these elements create a built-in mechanism for the gradual progression of difficulty.

Play also serves as a safe space for self-directed, trial-and-error learning with high tolerance of errors, as children can make mistakes without the fear of failure. Ossmy et al. [207] demonstrated exceptional tolerance for errors in infants while they are learning to walk. Infants frequently fall while learning to walk, but these trivial events do not hinder ongoing ML and exploration. This remarkable tolerance typically decreases at later developmental stages [207]. PA-play naturally embeds different learning styles and strategies. To elaborate, social play involving peer interactions, group dynamics, collaborations, and turn taking creates a rich context for observational learning, feedback exchange, co-regulation, and shared discovery. These social and environmental influences are strongly emphasized in the ML literature, where motor development is closely connected to contextual social factors in children [107]. Similarly, PA-play allows children to benefit from both implicit and explicit learning. They receive direct or indirect guidance and feedback from each other using gestures, metaphors, modeling, and shared action. However, these learning opportunities and elements from various ML strategies do not appear as structured opportunities. Instead, they emerge as flexible, overlapping components that adapt fluidly to the dynamic context of play.

Despite this overlap between PA-play and ML elements, the synthesis also highlighted that some aspects of PA-play do not fully align with some of the ML elements. Play is inherently fluid and allows for frequent shifts. For example, children could spontaneously enter and exit activities and divert their attention to different elements within the play. Unlike highly structured practice involving definite practice schedules, modes of instruction, and feedback, PA-play is not fixed nor structured. Therefore, PA-play in the context of ML is best understood as complementary to more structured, adult-guided motor practice, where elements like task-specific training, clear feedback, and progressive challenge are systematically applied. It means that attention should be drawn to incorporate opportunities for free, unstructured PA-play within guided, structured motor practice. This approach maintains the autonomy and enjoyment that characterize free play while simultaneously providing the scaffolding necessary to learn/refine motor skills. Together, these elements optimize skill acquisition and the transfer of motor skills to everyday, real-world contexts.

Lastly, we recommend extending recent work by Serino and Ossmy [208] on the importance of free play for cognitive development to ML. It is equally important to conceptualize ML as a continuous, dynamic process that emerges from rich, varied real-world experiences embedded in everyday play. Capturing developmental processes as they unfold naturally within the evolving, embodied, and socially scaffolded environment of play offers a more holistic perspective on skill acquisition. It also provides a causally informative lens for understanding how these skills develop in children.

### 4.2. Physically Active Play as a Context for Motor Learning in Children with Developmental Coordination Disorder

The synthesis suggests that children with DCD have less efficient ML processes compared with their TD peers, often characterized by slower acquisition, increased effort, greater performance variability, and reduced adaptability. Across the studies, there has been sustained interest in identifying effective motor interventions that may support learning in this population. However, there is no definitive consensus on how best to implement these ML elements across diverse contexts. This is due, in part, to the heterogeneity of the DCD population and variations in study design, ranging from controlled laboratory tasks to more ecologically valid settings [95,149,150,161,209,210,211,212,213,214]. The literature generally highlights the potential value of interventions grounded in ML principles, including repeated practice, progressive increases in task and environmental complexity, and the provision of clear guidance and timely feedback [18,22,147,215]. In addition, some studies suggest that interventions should combine task-specific training with real-life activities that emphasize physical activity, including PA-play [22,151]. This is driven by the understanding that a holistic approach to ML results in skill acquisition that is effectively generalized to everyday participation of the child [140].

Despite this, and even with the recent gradual integration of playful elements into ML studies (e.g., through video games) [193,195], our synthesis reveals a notable gap in the existing research at the intersection of ML and PA-play in children with DCD. To elaborate, most existing studies examine ML in controlled, isolated settings (e.g., tracing, aiming, or key-press tasks) that do not capture the dynamic, iterative nature of learning in real-life play contexts. While some research has integrated playful or whole-body activities [192,193,216], the study of ML as it naturally unfolds during PA-play remains scarce. Furthermore, lab-based tasks may reflect a child’s *capacity* or what they are capable of under ideal, controlled conditions. However, they often do not reflect *performance* or what children can actually do in different settings, ranging from home to school to the community.

The synthesis also highlights the reduced participation of children with DCD in PA-play, often stemming from motor challenges, low motivation, or social exclusion—factors that contribute to a persistent activity deficit or negative cycle [197,199]. Disrupting this negative cycle in children with DCD requires interventions that are both timely and attuned to how ML naturally unfolds in everyday life. PA-play may offer opportunities for children to explore movement, experiment with motor strategies, synchronize actions with peers, and receive intrinsic and social feedback. Given the limited availability of DCD-specific studies examining PA-play in relation to ML, the synthesis is necessarily inferential and draws on indirect conceptual connections rather than direct empirical evidence. Therefore, it is important to deepen the research on ML within the context of play. Understanding these natural learning processes is essential to designing interventions that are both ecologically valid and developmentally appropriate for children with DCD.

### 4.3. Clinical Directions

The current findings highlight that PA-play may represent a promising complementary context for supporting the learning and refining of motor skills in children, including those with DCD. Potential directions to support the thoughtful use of PA-play in clinical practice include integrating play-based evaluations with enriched environments and collaborating with families, educators, and community programs to promote meaningful and developmentally appropriate opportunities for active play. Observing children’s everyday play and engaging them and their caregivers in reflective dialogue about play experiences and perspectives can provide insights into children’s interests, motivations, and social dynamics, informing the design of activities that foster autonomy, meaningful choice, and social engagement [26,28,53,92,217,218,219].

Embedding play elements within therapy sessions, both individual and group, should be viewed as a central, developmentally appropriate process in its own, rather than merely a means to an end [25,32,125,220]. Therapeutic activities should be aligned to the child’s age, interests, and ML needs. For example, during the early stages of learning, the focus might be on trial-and-error learning opportunities to build confidence and foster peer collaborations. As learning progresses, encouraging independence and increasing task complexity will be important [178,221]. Expanding opportunities through inclusive, developmentally tailored play spaces can support skill acquisition, confidence, independence, and peer interaction [25,218].

Fostering supportive environments beyond therapy sessions can further enrich opportunities for PA-play. Collaboration with families, educators, and community programs can further reinforce motor-rich play in daily routines, enhancing the generalization of skills beyond the therapeutic setting. Providing guidance and tools will empower caregivers to support movement and motor experiences at home, in the community, and in local play spaces [222,223].

At a broader level, supporting accessible environments, integrating play-based approaches in early education, and providing tools to identify children at risk for reduced play participation can sustain engagement and promote lifelong motor competence, particularly in children with neurodevelopmental disorders such as DCD [224].

## 5. Limitations and Future Directions

Some of the limitations of the current study stem from the conceptual synthesis approach employed. Unlike a systematic review, which typically seeks exhaustive and reproducible extraction of all the relevant literature, this synthesis prioritized conceptual integration over comprehensive coverage and reproducibility. Consequently, a numerical reporting of records identified, screened, and included at each stage of the search is not provided; only the number of studies included in each stream. These features reflect a deliberate methodological choice driven by the broad, cross-disciplinary nature of the field and this study’s objectives, which prioritize conceptual integration over comprehensive coverage. Additionally, the inclusion of a mix of source types, including non-empirical sources, is consistent with this approach, allowing us to capture a broader, more integrative range of theoretical, clinical, pedagogical, and practical perspectives that contribute to understanding conceptual intersections across domains. However, this approach also means that some empirical studies were not included, and the synthesis was shaped by the conceptual nature of the research questions rather than being purely outcome-driven and interpretive in nature.

The use of a snowballing search strategy and relying on the authors’ clinical and research experience while drawing inferences (e.g., alignment between PA-play and ML) could introduce an element of subjectivity in the search and synthesis process. Additionally, the use of an organizational structure derived from a specific clinical perspective on ML, rather than a broader conceptual framework, represents a limitation in terms of the contextual framing of ML.

Future research could build upon this conceptual foundation by conducting more focused empirical studies or systematic reviews to further explore evidence-based relationships between PA-play and ML, especially in children with DCD. We were unable to examine all layers and nuances of each domain for the current narrative review. For example, in the context of PA-play, the analysis did not differentiate among play types (e.g., structured vs. unstructured, indoor vs. outdoor, and naturalistic vs. laboratory), which may have influenced the specificity of the findings. Additionally, age is one of the key factors in the development and progression of ML [98]; however, the synthesis did not categorize findings by age group. Future research can address these limitations by conducting empirical studies and systematic reviews that focus on more specific questions, allowing for in-depth subgroup analysis and exploring additional dimensions of PA-play and ML. This can inform developmental theory on motor development and can support the development of play-based approaches within educational and clinical settings.

## 6. Conclusions

PA-play provides a developmentally appropriate context that is consistent with some of the principles of ML in children. It aligns with ML elements, such as engagement and repetition, and introduces progression in a way that resonates with children’s natural inclinations. Consequently, PA-play, along with structured motor practice, can drive the development and refinement of motor skills in children. Stakeholders, including therapists, educators, and caregivers, should consider incorporating play-based activities across diverse environments. While these approaches may benefit children with neurodevelopmental conditions such as DCD, it is important to note that much of the existing evidence is drawn from neurotypical populations. Drawing on a conceptual synthesis rather than direct empirical testing or a systematic review, this work highlights potential directions for empirical exploration and practical application. It provides a structured foundation for future research to further explore how ML unfolds and evolves within the naturalistic context of PA-play, including examining specific elements of ML in PA-play; the influence of various types of play, focusing on age groups; and evaluating impact across different populations.

## Figures and Tables

**Table 1:** Alignment of physically active play with motor learning elements in typically developing children.

ML Elements	ML Trends in TD Children	Alignment Between PA-Play and ML Elements
**(a) General Learning Concepts**		
**Stages of skill acquisition**	Children go through different stages while acquiring new motor skills. However, children often operate in the novice stage, as they continuously acquire new skills and are exposed to new tasks/environments [19,69].	Children can be at different stages of motor skill learning across various skills demonstrated during play [83]. Note: Unlike Fitts and Posner’s stages of learning, contemporary theories such as dynamical systems and ecological theories emphasize focusing on the process of learning rather than the stages [84].
**Meaningful, salient goals**	Tasks that are salient and meaningful to children more effectively support ML by fostering a well-integrated person–task–environment triad [19].	Play is deeply meaningful to the child; it is self-chosen, autotelic and central to daily life [28,31,32,37,39,55,73,85,86].
**Active engagement/problem solving**	Active engagement of the learner in discovering motor solutions is essential to achieving task goals and promoting ML [19].	Play promotes deep, sustained, and immersive engagement [32]. Free play actively engages children’s senses by offering rich sensory experiences [87]. As it is both curiosity- and challenge-driven, it naturally supports creative thinking and problem solving [28,31,52,79,88,89,90].
**Practice challenge and progression of task difficulty**	Effective ML involves exposing children to the ‘just-right’ challenge with increasing demands to support skill development and sustained engagement [14,91].	Play naturally offers varying task difficulties. Progressions arise from self-direction and external factors like peer interaction, turn taking, game rules, and environmental cues [28,29,39,55,92].
**Learning mechanism**	Implicit learning is present early on and tends to be stable from early childhood to adolescence [93], with some studies suggesting a peak during late childhood/early adolescence [94]. Conversely, explicit learning improves with cognitive maturation, particularly with gains in working memory. Systematic reviews indicate that implicit and explicit approaches could be equally effective across different age groups [95]; instead, effectiveness may vary based on a child’s individual motor abilities and task constraints [96].	Play involves both types of learning; children can learn through doing, with or without explicit verbal explanation [39].
**Classification of motor skills**	The classification of motor skills or the type of task (e.g., gross/fine, open/closed, and discrete/continuous/serial) significantly influences ML in children [69]. For example, task complexity impacts the structure of training, including instruction, practice schedules, and feedback strategies [23].	Play is inherently versatile; children practice diverse skills that vary widely and are not structured by a specific motor skill classification system [31,83].
**(b) Practice Variables**		
**Amount of practice (dosage)/number of repetitions**	Children require more extensive, repeated practice over extended periods than adults to achieve effective ML and memory consolidation [19,97,98].	Play provides natural, engaging opportunities for motor practice, where deep involvement and enjoyment promote repetition without perceived effort [29,31,35,37,55,79,83,99]. Children can practice for hours [92]. However, play is usually flexible, fluctuating, with or without frequent rest periods [34], therefore, it might not provide enough repetition to support effective ML alone [23,98].
**Practice schedule**	Blocked or mixed practice has less contextual interference and so may be more beneficial for younger or less skilled learners performing complex tasks, whereas random practice, associated with high contextual interference, appears to benefit only older children (10–12 years), likely due to more mature cognitive capacities [19,100,101]. However, findings remain mixed, and no consistent advantage for any single practice schedule has been established [69,102].	Children do not follow a specific practice schedule during play.
**Practice variability**	Early in the learning process, constant practice is ideal to support learning and movement stabilization. On the other hand, varied practice is ideal to promote adaptability and transfer in later stages of learning [101].	Play predominantly offers a variable context for practice, where children engage in a wide range of self-initiated actions and adapt their movement parameters in response to constantly changing and unpredictable external constraints [35,37,51,83,103,104].
**Task breakdown**	Whole-task practice generally yields better skill performance and movement quality, especially in real-world contexts, while part-task practice may aid early learning. Young children benefit more from part-task practice and older children from whole-task practice, reflecting developmental differences in neural maturation, coordination, and learning capacity [69,105].	Children’s play flexibly blends part and whole practice, shifting between practicing single movements and integrated complex movements [28,99].
**Specificity**	As per the specificity of practice principle, practice closely matching real-world performance conditions enhances transfer regardless of the age of the child [19].	During play, children engage in meaningful, self-selected activities that are aligned with their individual interests and preferences, hence supporting the specificity of practice principle [31,106]. These play activities often mirror the movement patterns, contexts, and challenges encountered in daily life. This alignment enhances the relevance of practice and supports the transferability of learned motor skills to real-world tasks.
**Positive reinforcement**	Positive reinforcement supports ML, especially when it is aligned with the child’s interests and preferences [107].	Positive emotion, enjoyment, and peer interactions are natural positive reinforcers for children during play [32,40,53,55,83,99].
**Feedback**	The effectiveness of feedback during ML is influenced by factors such as type, frequency, timing, and mode of delivery [108]. For example, higher feedback frequency generally supports children’s ML, particularly in early stages [108]. The effectiveness of fading feedback appears to depend on both task complexity and developmental level, such that complex tasks and younger children might benefit from a gradual reduction in feedback compared with easier tasks and adults [109,110]. Limited evidence was found for the effect of visual feedback compared with verbal feedback [111]. Augmented and motivational feedback, especially when timely, positive, and self-controlled, can enhance both performance and perceived competence [112,113,114].	In play, feedback occurs naturally and fluidly within the flow of the activity. Children generate feedback through self-assessment, as well as peer interactions, which offer both verbal and non-verbal cues to guide performance [29,87,115].
**(c) Task Presentation**		
**Mode of instructions**	For younger children, visual instructions and modeling are more effective than verbal instructions, as they rely on visuo-spatial memory. As children’s cognitive abilities advance, especially executive skills and memory, they can process complex instructions, retain multiple steps, and apply rules. Hence, enabling the use of more integrated and complex teaching approaches [84,116].	Play involves both visual and verbal modes of instruction. Active games with defined roles embed instructional elements within playful contexts [39].
**Instruction’s direction of attention**	Both internal and external focus instructions can enhance ML. The impact of attentional focus during instruction depends on the timing, framing, developmental stage, and individual differences [117]. Studies show mixed effects of attentional focus, with some demonstrating that an external focus enhances retention and transfer of skills more than an internal focus, though results are inconsistent, and effects may be short-lived [19,118,119,120].	During play, especially with peers or adults, children attend to instructions using both internal and external focus [29].
**(d) Psychological dimension**		
**Intrinsic motivation** **and related affective and social–cognitive factors**	Motivation plays a key role in ML in children, such that practice under motivated conditions promotes deeper involvement, sustained attention and focus, enhanced information processing, and memory consolidation [19,23]. Motivation is closely linked to affective social–emotional factors (positive affect, satisfaction, and enjoyment) and social–cognitive factors (autonomy and control, self-efficacy, and self-perception) [107,121]. Granting children autonomy and control over their learning process significantly enhances their intrinsic motivation [113,122,123]. Similarly, higher perceived competence can boost self-efficacy and motivation for ML in children, and this in turn can further reinforce self-efficacy and perceived competence [107,121].	PA-play naturally embeds positive affective and social–cognitive factors related to intrinsic motivation [33,52,73]. It supports emotional well-being, attention, and affective social competence with social affiliation and cooperation [26,52]. Children describe play as joyful and exciting, often associating it with freedom, choice, and autonomy [32,53,92,124]. Additionally, play helps build self-efficacy and confidence, as it is low-stakes, highly flexible, embraces mistakes, and is a failure-tolerant environment [28,35,36,86,88,99,103,125].
**(e) Learning Strategies**		
**Trial-and-error learning**	ML in early childhood is fundamentally shaped by trial-and-error learning, emphasizing the active participation of the learner in the learning process. Children engage in an experiential and iterative process of countless repetitions of diverse movement patterns, and receive real-time feedback, which further guides adjustments and refinements of movements [17,19,23,84,98].	Trial and error are pivotal to PA-play, providing both intentional and incidental learning opportunities to explore, experiment, and discover new motor variations and solutions through iterative cycles of trying, observing outcomes, and refining actions [73,88]. In this safe, low-stakes, and flexible environment, children are free to take risks, make mistakes, and learn from their mistakes [31,36,88,99,126].
**Discovery learning**	Some evidence suggests that guided discovery learning (i.e., structured constraints and scaffolding) enhances children’s ML by promoting self-organized exploration and deeper active engagement, leading to more effective skill development [127].	Playful contexts encourage exploration and discovery [51,73,104]. PA-play encourages self-initiated discovery, often in more spontaneous ways than guided discovery. Yet, some movement-based, problem-solving games naturally implement discovery learning by combining structured constraints with an open-ended format. While rules provide meaningful motor challenges and guide the activity, the solution path is not predetermined, allowing children to actively explore, experiment, and find their own movement solutions [128].
**Observational learning**	Observational learning plays a key role in children’s ML, enabling them to learn new skills by watching and internalizing the actions of others [23]. Research suggests that children can effectively learn motor skills through observation, even with minimal instruction [129]. They benefit from observing expert models for accurate demonstrations, novice models for common errors and learning processes, and self-observation for self-awareness and motivation [23,130].	Play serves as a powerful context for observational learning, providing children with abundant, natural opportunities to observe and imitate their peers. Through this process, they gain insights into new and shared motor affordances, expand their motor repertoire, and choose whom to model based on others’ behaviors and reactions [28,29,32,103]. For older children, observing others can trigger feelings of competition and comparison. These dynamics may arise during the flow of play, where emotional fluctuations such as shifts in cooperation, competition, and reactions to success or failure can impact the observational learning process.
**Motor imagery**	Although limited, current evidence suggests that motor imagery enhances children’s ML and performance, especially when combined with physical practice, particularly for complex skills [131]. Young children tend to rely on physical practice, but as they grow, the ability to use motor imagery develops and improves through childhood and adolescence [131,132].	During play, young children often visualize themselves performing actions, such as ‘flying like a bird’ or ‘driving a race car,’ which actively connects motor actions with imagination [133]. However, this is usually done in the form of a mental image or visual imagination, as children before 6 years of age cannot evoke motor imagery without engaging in real motor activity [133,134].
**Errorless learning**	Some research suggests errorless learning benefits various populations, including children. Most studies focus on children with atypical development; however, similar benefits are seen in TD children, especially in early stages of learning. Errorless learning may enhance motivation and mastery while reducing social comparison pressures, thereby highlighting the importance of early success experiences [96,135,136].	Play does not specifically promote or embed this strategy.
**Analogy learning**	Analogy learning may help children, particularly novice learners who are acquiring complex motor skills, as it reduces cognitive processing demands [137,138]. Metaphorical instructions enhance children’s recall of self-generated motor representations in the absence of external cues, without affecting movement quality [139].	Play promotes analogical thinking by helping children connect their sensory–motor experiences to abstract concepts, often through imitating familiar roles and actions. For example, during pretend play, children use real-life objects and actions to represent different roles or scenarios. They experiment with the meanings and rules of the world while exploring various ideas, emotions, and relationships [126].

Note: TD = typically developing; ML = motor learning; PA-play = physically active play.

**Table 2:** Motor learning trends in children with Developmental Coordination Disorder.

ML Elements	ML Trends in Children with DCD	PA-Play-Related Notes *Based on Typical Development*
**(a) General Learning Concepts**		
**Stages of skill acquisition**	Children with DCD typically progress more slowly and effortfully through stages of skill acquisition, often requiring more time, support, and repetition compared with TD peers. They may use compensatory but less efficient strategies to achieve task goals, although these solutions are often less efficient [18,131]. In the initial stage, they often need more time, repetition, and structured support due to difficulties in processing instructions, forming internal models of movement, understanding movement goals, and initiating planned actions [18,140,141].	
**Meaningful/salient goals**	Setting goals that are meaningful to the child and aligned with their interests is at the core of many ML task-based interventions for children with DCD. These goals reflect what is important to the child within their natural environments and daily routines, thereby enhancing relevance, motivation, and functional engagement [140,142].	PA-play in childhood is inherently meaningful and carries profound significance, particularly when it is customized to the child’s interests and preferences.
**Active engagement/problem solving**	Active engagement and problem solving are essential to ML, but children with DCD may struggle with active engagement due to motor difficulties, low self-efficacy, and contextual barriers (e.g., limited support or negative attitudes) [18,22,143,144,145]. Thus, providing structured opportunities for problem solving is crucial to supporting learning in this population [18,22,143,144,145].	
**Practice challenge and progression of task difficulty**	Effective ML in DCD relies on carefully graded task difficulty. Initially, simple tasks with high levels of assistance are helpful, which could then be progressed as skill develops [146]. This ML element is a core component of broader task-oriented interventions for children with DCD such as Neuromotor Task Training [140,144,145,146,147].	PA-play naturally offers intrinsic progression, with task difficulty evolving through self-direction and external factors. To ensure the appropriate level of challenge, guided PA-play can be integrated, tailoring the difficulty to the child’s abilities and facilitating a gradual increase in task demands for effective and engaging learning
**Learning mechanism**	There is no clear consensus regarding implicit/explicit learning mechanisms in children with DCD [148,149,150]. Some studies reported impaired implicit learning in DCD [148,149], while others suggest it remains intact [150].	
**Classification of motor skills**	Classifying motor skills is essential in DCD, as learning difficulties often vary based on task complexity. Differences from peers are often more evident on complex tasks, whereas simpler tasks tend to show comparable outcomes [146], hence underscoring the importance of aligning task type with the child’s motor profile [151,152].	
**(b) Practice Variables**		
**Amount of practice (dosage)/number of repetitions**	Children with DCD require more repetitions and extended practice time compared with TD children to achieve satisfactory performance levels, particularly for complex or highly planned motor tasks [18,22,143,144,145,153,154,155,156,157]. While repetitive practice is essential, repetition alone is not sufficient unless combined with problem-solving opportunities and task-specific training [18,22,143,144,145].	PA-play can provide opportunities for repeated practice through intrinsic motivation and repeated exposure to trials.
**Practice schedule**	There is some evidence to support the use of random practice (higher contextual interference) compared with blocked practice in children with DCD. However, further research is needed to validate the findings [158].	
**Practice variability**	Children with DCD can benefit from structured variability in practice to promote generalization of motor skills; however, the degree of variation must be carefully calibrated to avoid overwhelming their motor system [159,160]. Notably, Bonny et al. [161] reported no significant difference between varied and constant practice, highlighting the need for further research to clarify optimal practice schedules for this population.	PA-play can offer practice variability by allowing children to engage in diverse, dynamic tasks.
**Task breakdown**	One of the DCD intervention approaches, Neuromotor Task Training, employs task breakdown to identify and target specific components of motor tasks that are challenging for the child, thus enabling structured, progressive practice tailored to the individual needs and functional goals of the child [142].	
**Specificity**	Apart from the Neuromotor Task Training, limited research exists examining the use of specificity in the DCD population.	
**Positive reinforcement**	Tangible positive reinforcers such as food, preferred activity, social praise, and breaks are commonly used in pediatric rehabilitation. But their efficacy has not been systematically explored in the DCD population.	Play that aligns with the child’s preferences and abilities offers reinforcement through enjoyment and achievement.
**Feedback**	Children with DCD show deficits in internal feedback (e.g., predictive control and self-monitoring), rely more on external and online visual feedback, and need more time to process feedback compared with TD peers [162,163]. This often results in an over-reliance on external forms of feedback, particularly during movement execution, which may limit the development of more efficient internal feedback systems [18]. Feedback is beneficial for children with DCD, but no specific type shows conclusive superiority (e.g., internal vs. external attentional focus, knowledge of results vs. performance, or technology-based feedback) [164]. Both internal and external focus can support ML in DCD, though individual responses vary. Self-controlled or technology-supported feedback delivered at higher frequencies may enhance engagement and retention [165]. Technology-assisted and augmented visual feedback show promising results [144,157]. Feedback effectiveness likely depends on factors like age, motor skill level, visuo-spatial memory, and task type/complexity [166].	Feedback during PA-play can be supportive, but its uncontrolled nature makes the impact uncertain, highlighting the need for adult-guided play.
**(c) Task Presentation**		
**Mode of instructions**	The effectiveness of specific instructional modes in children with DCD varies depending on the child’s individual characteristics and the nature of the task [166,167]. To facilitate task understanding and execution, instruction often requires reducing cognitive load by simplifying task demands and modifying the mode of delivery. This can include using visual supports, breaking complex tasks into manageable steps, and adopting less stringent or more flexible task rules.	
**Instruction’s direction of attention**	Limited evidence exploring the use of attentional focus on ML in children with DCD exists, with Jarus et al. [168] indicating no differences between an internal and external focus of attention during a computerized tracking task.	
**(d) Psychological dimension**		
**Intrinsic motivation** **and related affective and social–cognitive factors**	Children with DCD are at risk of a negative motivational cycle, where persistent motor challenges reduce enjoyment, increase apprehension, and lower perceived competence and self-efficacy. This in turn further reduces motivation, limits engagement, and negatively affects physical activity-related cognitions and social participation [18,169,170,171,172].	Tailored PA-play, based on the child’s preferences, has the potential to enhance motivation and self-efficacy by promoting success through non-competitive or guided play. Additionally, it supports social learning through group activities, collaborative problem solving, and shared goals.
**(e) Learning Strategies**		
**Trial-and-error learning**	Children with DCD often show limited effectiveness in trial-and-error learning due to difficulties in error detection, correction, and feedback integration. As a result, they typically require a longer exploration phase and more repetitions and guidance to discover effective movement strategies. These challenges may stem from deficits in internal modeling and sensory prediction, which impair their ability to learn efficiently from movement errors and adapt accordingly [173].	PA-play can support trial-and-error learning by providing a safe, iterative environment with structured guidance or games encouraging experimentation and error correction.
**Discovery learning**	Discovery learning is under-researched in the DCD population. However, guided forms of discovery (e.g., the Goal–Plan–Do–Check model from Cognitive Orientation to daily Occupational Performance, or CO-OP) have shown benefits, especially when compared with no intervention [174].	PA-play, especially outdoor play, offers a motor-rich environment that fosters discovery learning, allowing self-directed learning in a low-pressure setting.
**Observational learning**	Children with DCD show reduced effectiveness in observational learning, suggesting difficulties in using observed actions to support motor skill acquisition [173,175]. Combining action observation with motor imagery can enhance motor planning and performance in this population [176,177,178].	PA-play, especially in non-competitive, imitation-based games, offers children opportunities to observe and replicate motor actions at their own pace in a low-stakes environment.
**Motor imagery**	Motor imagery is often challenging for children with DCD, particularly for complex movements, likely due to impairments in internal modeling and mirror neuron activation [157,173,177,179,180]. Nonetheless, motor imagery improves with age [180], and motor imagery-based interventions, especially when combined with action observation, have demonstrated promising results in improving motor and daily functional skills in children with DCD [157,176,177,178].	
**Errorless learning**	Error-reduced learning has shown promising results in improving fundamental movement skills in children with DCD as well as self-perceived physical competence, particularly for object-control skills. Reducing errors during practice lowers working memory demands, an area known to be difficult in DCD [181]. However, evidence for errorless learning is limited in DCD.	
**Analogy learning**	Analogy learning could be effective for children with DCD; however, further research is needed [174].	

Note: ML = motor learning; DCD = developmental coordination disorder; TD = typically developing.

## Data Availability

No new data were created or analyzed in this study. Data sharing is not applicable to this article.
